# *Achromobacter* spp.: Emerging pathogens in the cystic fibrosis lung

**DOI:** 10.1371/journal.ppat.1013067

**Published:** 2025-04-22

**Authors:** Niladri Bhowmik, Reed M. Stubbendieck

**Affiliations:** Department of Microbiology and Molecular Genetics, Oklahoma State University, Stillwater, Oklahoma, United States of America; Tufts Univ School of Medicine, UNITED STATES OF AMERICA

## What are *Achromobacter* spp. and how do they infect the lungs of people with cystic fibrosis?

*Achromobacter* spp. (Phylum: *Pseudomonadota,* Class: *Betaproteobacteria,* Order: *Burkholderiales*, Family: *Alcaligenaceae*) are Gram-negative, strictly aerobic, non-spore forming, rod-shaped bacteria with peritrichous flagella, and are commonly found in soil and aquatic environments. The type species *Achromobacter xylosoxidans* was first described in 1971, as an isolate from human ear discharge, and the genus was validly published in 1981, with several species of the closely related *Alcaligenes* subsequently reclassified as *Achromobacter* [1–3]. These bacteria are increasingly isolated from people who are immunocompromised or individuals with cystic fibrosis (CF), raising globally from ~2%–3% between the 1990s and early 2000s to ~7%–10% by the early 2020s [4–8]. In CF patients, *Achromobacter* prevalence and infection varies based on age, and adults (≥18 years old) are more likely to be colonized [9]. Infections with *Achromobacter* spp. are frequently difficult to treat because these bacteria harbor numerous antibiotic resistance genes [10]. For example, *A. xylosoxidans* is frequently resistant to aminoglycosides, aztreonam, and cephalosporins, due to the presence of multidrug resistance pumps from the resistance-nodulation-cell division superfamily [11].

In CF, mutations in the CF transmembrane conductance regulator gene lead to accumulation of thick and viscous mucus in the lungs which serves as a substrate for colonization by *Achromobacter* and other microbes from the environment [12]. The sinuses or other body sites may be potential reservoirs for *Achromobacter*, as patients have been reported to become reinfected with the same strain after a bilateral lung transplant. However, it may also be possible that these individuals became reinfected from the same environmental source [5]. Furthermore, patient-to-patient transmission has been observed, as patients in close contact during prolonged periods can harbor strains of *A. xylosoxidans* with the same genotype [13]. Similar cases of transmission between CF patients have also been identified for other *Achromobacter* species using genetic distance measurements and epidemiological data [14].

## Which species of *Achromobacter* have been found to infect the lungs of people with CF?

The genus *Achromobacter* consists of 22 characterized and validly published species, among which 10 have been clinically isolated. Though the primary species associated with infection is *A. xylosoxidans*, many initial studies did not discriminate between this and other *Achromobacter* species. Identification of *Achromobacter* to the species level requires sequencing of the *nrdA* gene [15] or whole genome sequencing [14]*.* Other species that have been clinically isolated from CF patients are *Achromobacter aegrifaciens*, *Achromobacter animicus*, *Achromobacter dolens*, *Achromobacter insolitus*, *Achromobacter insuavis*, *Achromobacter marplatensis*, *Achromobacter mucicolens*, *Achromobacter pulmonis*, *Achromobacter ruhlandii*, and *Achromobacter spanius*. The prevalence of different *Achromobacter* species in CF patients varies based on geographical location. For example, *A. insuavis* has been isolated mostly from patients in Denmark and France, compared with the UK, Italy, and Argentina where the rate of its occurrence is low. In the USA, *A. dolens* is the most prevalent clinical isolate after *A. xylosoxidans*. In contrast, the occurrence of *A. dolens* is lower in the UK, Italy, and Denmark [9]. The higher prevalence of some *Achromobacter* species over others may signify variable environmental distribution [16].

## What are the clinical effects of *Achromobacter* on the lungs of people with CF?

Multiple studies from CF clinics globally have reported associations between infection with *Achromobacter* spp. and lung function decline (Table 1). Notably, initial case-control studies by Hansen and colleagues revealed that chronic *Achromobacter* infection leads to significant loss in respiratory function over time, as measured with spirometry using forced expiratory volume in 1 s (FEV_1_) [17,18]. Though these initial studies were limited with respect to sample size, an association between *Achromobacter* infection and FEV_1_ decline either compared with pre-infection or measured over the course of a chronic infection has been repeatedly reported in clinical analyses [7,19–22], including from an analysis of >2,000 people with CF and *Achromobacter* infection in Europe [22]. Furthermore, these analyses have found that patients with *Achromobacter* infection tend to have increased numbers of pulmonary exacerbations (PEx) [7,19–21,23], leading to more hospitalizations [21,23,24], and are more likely to be prescribed antibiotics [7,22,23].

**Table 1:** Associative studies supporting the role of *Achromobacter* spp. in disease in patients with CF.

Author, Year	Median Age (y), Interquartile Range [IQR]	Number of Patients Positive for *Achromobacter* (% Total)	Effect^1^	Association With Other Pathogens^2^^,^^3^
Hansen, 2006 [17]	15.7, [12.4–21.6]^4^	22 (7.7%)	↓ FEV_1_ over 3 y ↓ FVC over 3 y	
Hansen, 2010 [18]	20.4, [16.1–26.9]	14 (20.9%)	↓ FEV_1_ over 2 y ↑ GM-CSF, TNF-α detected	
Lambaise, 2011 [10]	21.1, [13.5–28.8]^4^	53 (17.6%)	No specific effect reported	↑ *Pa,* coinfection was not associated with increased FEV_1_ decline
Firmida, 2016 [24]	7.0, [4.0–18.0]^4^	47 (19.7%)	↑ Hospitalizations	↑ *Sa*
Edwards, 2017 [23]	24.0, [20.3–29.8]	34 (11.1%)	↑ Hospitalizations ↑ PEx ↑ Steroid administration	↑ *Sa*
Somayaji, 2017 [6]	18.2 [13.3–28.4]	88 (8.0%)	↑ Lung transplantation and death	
Recio, 2018 [19]	27.8, [19.2–36.4]^4^	25 (13.1%)	↓ FEV_1_ over 13 y ↑ PEx	↑ *Pa*, *Sa*
Tetart, 2019 [20]	23.5, [20.0–31.0]	36 (13.1%)	↓ FEV_1_ over 3 y ↓ Ventilatory Function ↑ PEx	*Pa*, coinfection associated with greater FEV_1_ decline
Marsac, 2021 [7]	11.5, [6.6–16.4]	45 (9.4%)	↓ FEV_1_ over 2 y ↑ Antibiotic administration ↑ PEx ↑ Steroid administration	↑ *Pa*, *Sa*
Sunman, 2022 [21]	7.7, [5.3–12]	37 (10.5%)	↓ FEV_1_ over 1 y ↑ Hospitalizations ↑ PEx	
Kerem, 2023 [22]	21.5, [14.0–29.5]	2,093 (5.4%)	↓ FEV_1_ ↑ Antibiotic administration ↑ Steroid administration	↑ NTM, *Sa*, Smc ↓ Bcc *Pa*, coinfection associated with greater FEV_1_ decline

PEx, pulmonary exacerbation; FEV_1_, forced expiratory volume in 1 s; FVC, expiratory forced vital capacity; GM-CSF, granulocyte-macrophage colony-stimulating factor; TNF-α, tumor necrosis factor alpha.

^1^ Up-arrows (↑) and down-arrows (↓) indicate that *Achromobacter* was associated with increased or decreased risk of the corresponding event, respectively.

^2^ Pathogens: *Burkholderia cepacia* complex, BCC; non-tuberculosis mycobacterium, NTM; *P. aeruginosa, Pa; S. maltophilia* complex, Smc.

^3^ Up-arrows (↑) and down-arrows (↓) indicate that *Achromobacter* was associated with increased or decreased co-infection with the corresponding pathogen, respectively.

^4^ Estimated IQR based on reported ranges, assuming normal distribution where mean and median are identical.

## What is the effect of *Achromobacter* infection on inflammation?

*Achromobacter* infections are associated with a strong inflammatory response. People with CF that are infected by *Achromobacter* spp. exhibit higher serum levels of the proinflammatory cytokines granulocyte-macrophage colony stimulating factor (GM-CSF), interleukin 6 (IL-6), and tumor necrosis factor alpha (TNFα) [18]. Accordingly, these individuals are also more likely to be prescribed steroids [7,9,10]. Consistent with these findings, mice infected with *A. xylosoxidans* have elevated levels of the above factors and the proinflammatory interleukins 1α (IL-1α) and 1β (IL-1β) in bronchoalveolar lavage fluid [25,26]. Moreover, the lungs of infected mice show signs of a strong inflammatory response, including alveolar wall thickening, edema, and accumulation of infiltrating neutrophils and other polymorphonuclear cells [25,26]. The degree of lung tissue damage is correlated with levels of TNFα and the anti-inflammatory interleukin 10 (IL-10) [25].

Genes encoding type III secretion systems (T3SS) are common among *Achromobacter* spp. These needle-like systems are used by Gram-negative bacteria to inject toxic effectors directly into eukaryotic cells in a contact-dependent manner. The most-well characterization of these effectors in *Achromobacter* is AxoU, an effector with phospholipase activity [27]. In *in vitro* infection models, *A. xylosoxidans* adheres to macrophage and macrophage-like cells using a large RTX adhesin named ArtA and is internalized via phagocytosis [28]. Once internalized, *A. xylosoxidans* activates expression of *axoU* and other T3SS-associated genes, which are required for its cytotoxicity [27]. *Achromobacter insuavis* and *A. xylosoxidans* both use the T3SS to kill macrophages by inducing inflammosome-dependent pyroptosis, which promotes IL-6 production and inflammatory lung damage *in vivo* [29]. Pyroptosis occurs even in the absence of AxoU, suggesting that the T3SS also delivers other effectors. In mice, *A. xylosoxidans* spreads from the lungs into the bloodstream, but mutants that are unable to produce a functional T3SS apparatus fail to disseminate [26] and do not induce lung inflammation [29]. In addition to pyroptosis-mediated inflammation, *Achromobacter* spp. likely produce additional inflammatory factors. For instance, culture supernatants from *A. dolens*, *A. ruhlandii*, and *A. xylosoxidans* upregulate CD11b in monocytes and neutrophils, which induces IL-6 and interleukin-8 (IL-8) release from CF bronchial epithelial cells [30]. The specific identities and underlying mechanisms of the potential contact-independent inflammatory factors produced by *Achromobacter* spp. are currently unknown.

It is possible that some of these inflammatory factors are secondary metabolites called siderophores. Bacteria and other microbes use siderophores to scavenge ferric iron and other metal ions from their external environment [31]. Siderophores can also contribute to inflammation and virulence. For example, *Pseudomonas aeruginosa* produces the siderophore pyoverdine, which kills macrophages *in vitro*, and whose production levels are positively correlated with mortality in murine and worm infection models [32,33]. A recent survey has indicated that 85% and 90% of clinical isolates of *Achromobacter* spp. from CF and non-CF patients, respectively, produce siderophores. Clinical isolates also produce higher siderophore levels compared with environmental strains [34]. However, it is not currently known how siderophore production is related to *Achromobacter* spp. virulence *in vivo*.

## Do *Achromobacter* spp. interact with other CF pathogens during infection?

The lung microbiota in people with CF often comprises multiple bacterial and fungal species, including other pathogenic microbes. Individuals with chronic *Achromobacter* infection are frequently also co-infected with *P. aeruginosa* [7,10,19,22,35], *Staphylococcus aureus* [7,19,22–24], members of the *Stenotrophomonas maltophilia* complex [22], or nontuberculosis mycobacteria [22]. These co-infections can exacerbate lung function decline. For instance, people with CF that are co-infected with *Achromobacter* spp. and *P. aeruginosa* can have worse annual FEV_1_ decline when compared with people with mono-infections of either pathogen [20,22]. These effects could be due to an additive effect of infection with two pathogens or may be due specifically to a synergistic effect of co-infection. It is possible that interactions between *Achromobacter* spp. and *P. aeruginosa* modulate production of virulence factors by one or both pathogens. For instance, a recent study determined co-culture of one *A. xylosoxidans* strain decreases *P. aeruginosa* biofilm formation, motility, siderophore production, and mortality in a zebrafish embryo infection model, while another strain had little effect [36]*.* It is important to note that in some studies FEV_1_ decline in CF patients infected with *Achromobacter* spp. occurs independently of *P. aeruginosa* infection [19] and other studies have not found any specific effect on respiratory function associated with *Achromobacter* infection [6,10].

*Achromobacter* spp. engage in competitive and cooperative interactions with other CF lung microbes. Co-culture assays with clinical *A. xylosoxidans* and *P. aeruginosa* isolates revealed extensive interspecies interactions, with nearly half of the combinations resulting in alterations to growth, motility, or pigment production in *P. aeruginosa*, both positively and negatively [37]. The specific mechanisms used by *Achromobacter* spp. to mediate these interactions are unknown but may involve production of secondary metabolites or the type VI secretion system (T6SS). We highlighted siderophores as secondary metabolites that may act as virulence factors, but these same metabolites are frequently used by bacteria to sequester metals from their competitors [31]. Furthermore, a CF sputum isolate of *A. xylosoxidans* produces the secondary metabolite lasso peptide achromodin-1 [38]. Achromodin-1 is an RNA polymerase inhibitor that has a narrow activity spectrum, affecting only *A. pulmonis* and an *Escherichia coli* mutant with increased outer membrane permeability. This limited activity spectrum suggests that achromodin-1 may facilitate competition between closely related *Achromobacter* spp. co-infecting the lung [38]. In addition, most isolates of *Achromobacter* spp. encode components for a T6SS, designated as TAX-1 [39]. The T6SS apparatus has components with structural homology to bacteriophage tail proteins and is used by Gram-negative bacteria to deliver toxic proteins into adjacent cells [31]. One of the TAX-1 genes is a predicted effector with phospholipase D (PLD) activity. Strains of *A. xylosoxidans* can kill *E. coli* and *P. aeruginosa* in a T6SS-dependent manner, presumably using the PLD effector or another effector protein [39]. Proteomic analysis of the secretome of *A. xylosoxidans* revealed that T6SS components are produced in chemically defined medium that mimics the nutrient-limiting conditions of the CF lung and a T6SS component has been detected directly in sputa from people with CF, suggesting a role for this system *in vivo* [39].

## Future directions

*Achromobacter* spp. are recently emerging and relatively understudied pathogens in the lungs of people with CF, where they are associated with lung function decline, increased PEx, and increased use of antibiotics and steroids. There is a diverse spectrum of *Achromobacter* species and knowledge regarding their clinical relevance is still limited. Some of these species are more prevalent in clinical settings, though the mechanisms underlying this phenomenon remain unknown. These bacteria provoke inflammation through mechanisms such as T3SS and other virulence factors, potentially including siderophores. Furthermore, *Achromobacter* spp. interact with other members of the CF lung microbiota, potentially influencing disease progression. However, the mechanisms underlying these interactions remain poorly understood, as does their impact on the composition of the microbiota and disease dynamics in the lungs of people with CF. *Achromobacter* spp. are also implicated in other chronic infections, including non-CF bronchiectasis [40], and have been recovered alongside *P. aeruginosa* in green-nail syndrome [41] and *S. aureus* in osteomyelitis [42], though whether their interactions in these environments parallel those observed in the CF lung is unknown. Investigating interactions between *Achromobacter* spp. and other microbes may lead to better understanding of the complex interactions among chronic infection microbiomes and lead to the identification of novel therapeutics.
